# Gut microbiome patterns correlate with higher postoperative complication rates after pancreatic surgery

**DOI:** 10.1186/s12866-019-1399-5

**Published:** 2019-02-18

**Authors:** Felix C. F. Schmitt, Thorsten Brenner, Florian Uhle, Svenja Loesch, Thilo Hackert, Alexis Ulrich, Stefan Hofer, Alexander H. Dalpke, Markus A. Weigand, Sébastien Boutin

**Affiliations:** 1 Department of Anesthesiology, Heidelberg University Hospital, 110, Im Neuenheimer Feld, D-69120 Heidelberg, Germany; 2 Department of General, Visceral and Transplant Surgery, Heidelberg University Hospital, Heidelberg, Germany; 3 Department of Anesthesiology, Kaiserslautern Westpfalz Hospital, Kaiserslautern, Germany; 4 Department of Infectious Diseases, Medical Microbiology and Hygiene, Heidelberg University Hospital, Heidelberg, Germany; 5 Translational Lung Research Center Heidelberg (TLRC), German Center for Lung Research (DZL), Heidelberg, Germany; 6 Institute of Medical Microbiology and Hygiene, Technical University Dresden, Dresden, Germany

**Keywords:** Pancreas, Postoperative complications, Gut microbiome, 16S RNA gene sequencing, Inflammation, Sepsis

## Abstract

**Background:**

Postoperative complications are of great relevance in daily clinical practice, and the gut microbiome might play an important role by preventing pathogens from crossing the intestinal barrier. The two aims of this prospective clinical pilot study were: (1) to examine changes in the gut microbiome following pancreatic surgery, and (2) to correlate these changes with the postoperative course of the patient.

**Results:**

In total, 116 stool samples of 32 patients undergoing pancreatic surgery were analysed by 16S-rRNA gene next-generation sequencing*.* One sample per patient was collected preoperatively in order to determine the baseline gut microbiome without exposure to surgical stress and/or antibiotic use. At least two further samples were obtained within the first 10 days following the surgical procedure to observe longitudinal changes in the gut microbiome. Whenever complications occurred, further samples were examined.

Based on the structure of the gut microbiome, the samples could be allocated into three different microbial communities (A, B and C). Community B showed an increase in *Akkermansia*, *Enterobacteriaceae* and *Bacteroidales* as well as a decrease in *Lachnospiraceae*, *Prevotella* and *Bacteroides*. Patients showing a microbial composition resembling community B at least once during the observation period were found to have a significantly higher risk for developing postoperative complications (B vs. A, odds ratio = 4.96, *p* < 0.01**; B vs. C, odds ratio = 2.89, *p* = 0.019*).

**Conclusions:**

The structure of the gut microbiome is associated with the development of postoperative complications.

**Electronic supplementary material:**

The online version of this article (10.1186/s12866-019-1399-5) contains supplementary material, which is available to authorized users.

## Background

Despite the constant improvement in operation techniques, intensive care and standardised antibiotic use, postoperative complications are still a significant problem in daily clinical practice. Over the past years, the use of next-generation sequencing (NGS) has changed our perception and understanding of the structure and function of the microbiome in various organ systems. The gut microbiome has been shown to play an important role in preventing opportunistic and nosocomial infections, which are becoming more frequent due to widespread antibiotic use [1–3]. Under normal conditions, specific members of the normally abundant gut microbiota interact with low abundance pathobiota and prevent them from crossing the intestinal barrier. At the same time, the gut microbiome composition appears to reflect the efficiency of our immune system to react to invasive pathogens [4–6]. The use of NGS allows new insights into the complex composition of the intestinal flora and its reaction to external influences. Several studies have shown a correlation between the composition of the gut microbiome and certain diseases, such as obesity, irritable bowel syndrome and Crohn’s disease, as well as disease severity [7–9]. Additionally, the gut microbiome appears to change dramatically in critically ill patients. A significant decrease in bacterial diversity together with a flare-up of problematic bacteria such as *Clostridium difficile* and vancomycin-resistant enterococci has been observed [10–16]. Moreover, it should be acknowledged that anti-infective treatment strategies (e.g., antibiotics and antimycotics) are able to aggravate microbial imbalance, paving the way for further surgical site infections [5]. Unfortunately, our ability to predict postoperative complications is limited [17], and the extent to which surgical stress and perioperative antibiotic use influence the gut microbiome are yet to be clarified. Similarly, we are still unclear as to whether changes in the microbiome can be used to predict complications during the postoperative clinical course. Thus, this prospective, observational, clinical study aimed to examine changes in the gut microbiome, as well as the consequences on the postoperative course, after pancreatic surgery. Our working hypothesis was that (1) the gut microbiome experiences major changes in the perioperative setting, and (2) a change in bacterial diversity or composition might have an impact on the incidence of postoperative complications.

## Results

### Patient characteristics

**Fig. 1:**
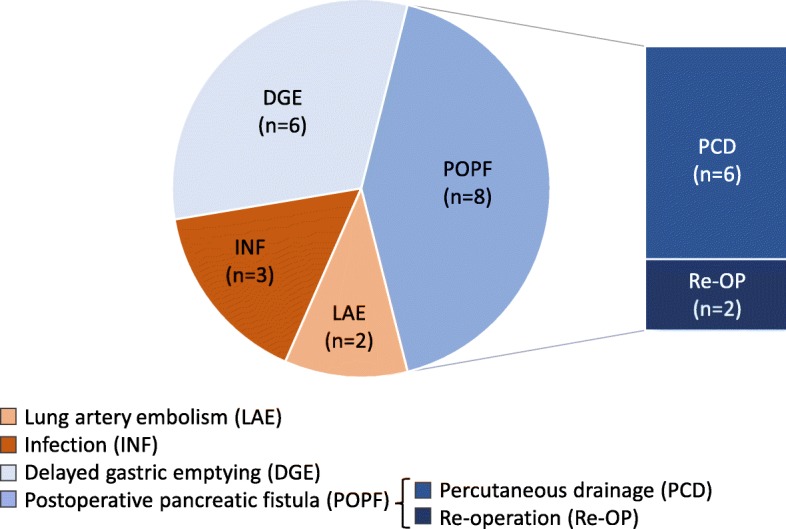
Postoperative complications, subdivided into two groups depending on whether they experienced medical (*n* = 5, reddish coloured) or surgical (*n* = 14, bluish coloured) complications

In total, 32 patients undergoing pancreatic surgery were included in the final evaluation, of whom 17 patients suffered postoperative complications and 15 had a non-complicated clinical course. Patients with a non-complicated clinical course were significantly younger and had a lower American Society of Anesthesiologists (ASA) status than those that suffered complications. Most clinical baseline characteristics prior to surgery (e.g., cardiovascular risk factors) as well as perioperative factors (surgery duration, type of operation and blood loss) showed no significant differences between the complicated and non-complicated groups. However, in the postoperative period, patients in the complicated group showed significantly increased C-reactive protein (CRP) levels, a higher leucocyte count, prolonged hospital stay and longer time in the intensive care unit (ICU) **(**Table 1**)**. The complicated group was further subdivided into two groups depending on whether they experienced medical (*n* = 5) or surgical (*n* = 14) complications. Two patients suffered from both a medical and surgical complication; therefore, both patients were allocated into both the medical and surgical group. Two patients in the medical group suffered from a lung artery embolism, requiring ICU treatment. Three patients displayed signs of an infection (e.g., urinary tract infection) and were treated with antibiotics. In the group of patients that experienced a surgical complication, six patients were treated with percutaneous drainage and two patients underwent re-operation due to a postoperative pancreatic fistula. Six patients were affected by delayed gastric emptying, whereas none of the patients suffered from postoperative bleeding. All 32 patients survived the 90-day observation period. For a better overview, Fig. 1 summarizes all postoperative complications.

**Table 1:** Patients’ characteristics

	Non-complicated	Medical complication	Surgical complication	All complications	*p*-value non-comp. vs. med. Comp.	*p*-value non-comp. vs. surg. Comp.	*p*-value non-comp. vs. all comp.
Number	15	5^1^	14^1^	17			
Age [years]	59.0 (50.0–63.5)	75.0 (75.0–75.0)	68.5 (63.3–74.0)	69.0 (62–75)	0.013*	0.009**	0.002**
ASA status	2.0 (2.0–2.0)	2.0 (2.0–3.0)	3.0 (2.0–3.0)	3.0 (2.0–3.0)	*0.327*	0.014*	0.021*
Cardiovascular risk factors							
Coronary heart disease	2 (13.3%)	1 (20.0%)	1 (7.1%)	1 (5.9%)	*0.718*	*0.584*	*0.801*
Diabetes	3 (20.0%)	2 (40.0%)	4 (28.6%)	5 (29.4%)	*0.371*	*0.590*	*0.447*
Arterial hypertension	5 (33.3%)	4 (80.0%)	6 (42.9%)	8 (47.1%)	*0.069*	*0.597*	*0.260*
Chronic renal failure	0 (0.0%)	0 (0.0%)	0 (0.0%)	0 (0.0%)	*1.000*	*1.000*	*1.000*
Primary disease							
Malignant	8 (53.3%)	4 (80.0%)	8 (57.1%)	11 (64.7%)	*0.292*	*0.837*	*0.563*
IPMN	2 (12.5%)	1 (20.0%)	6 (42.9%)	6 (35.3%)	*0.718*	*0.075*	*0.123*
Chronic pancreatitis	5 (33.3%)	0 (0.0%)	0 (0.0%)	0 (0.0%)	*0.136*	0.018*	0.006**
Operation							
Partial pancreaticoduodenectomy	3 (20.0%)	1 (20.0%)	3 (21.4%)	4 (23.5%)	*1.000*	*0.924*	*0.940*
Pylorus-preserving pancreas head resection	5 (33.3%)	3 (60.0%)	5 (35.7%)	6 (35.3%)	*0.292*	*0.893*	*0.601*
Total pancreatectomy	2 (13.3%)	1 (20.0%)	0 (0.0%)	1 (5.9%)	*0.718*	*0.157*	*0.410*
Distal pancreatectomy	3 (20.0%)	0 (0.0%)	3 (21.4%)	3 (17.7%)	*0.278*	*0.924*	*0.749*
Tumour enucleation	2 (13.3%)	0 (0.0%)	3 (21.4%)	3 (17.7%)	*0.389*	*0.564*	*0.841*
Surgery [min]	285.0 (187.5–345.0)	240.0 (240.0–330.0)	217.5 (153.8–337.5)	240.0 (165.0–330.0)	*0.726*	*0.444*	*0.639*
Blood loss^2^ [mL]	750.0 (500.0–1150.0)	700.0 (600.0–800.0)	600.0 (500.0–700.0)	600.0 (500.0–800.0)	*0.965*	*0.273*	*0.393*
Infusion^2^ [mL]	3500.0 (2500.0–4250.0)	3000.0 (3000.0–4500.0)	2750.0 (2000.0–3375.0)	3000.0 (2000.0–3500.0)	*0.756*	*0.186*	*0.352*
Transfusion^2^ [mL]	0.0 (0.0%)	0.0 (0.0%)	0.0 (0.0%)	0.0 (0.0%)	*1.000*	*1.000*	*1.000*
CRP^3^ [mg/L]	56.2 (36.4–114.8)	135.6 (79.9–151.9)	114.0 (76.9–160.7)	103.9 (74.4–153.0)	0.020*	0.004**	0.001**
Leucocytes^3^ [1/nL]	9.0 (7.9–10.3)	11.4 (9.0–17.4)	11.7 (76.9–160.9)	11.4 (8.3–14.0)	0.015*	0.025*	0.007**
Hospital stay							
In total [days]	10.0 (9.0–11.5)	21.0 (13.0–45.0)	27.0 (15.0–44.5)	21.0 (13.0–43.0)	0.007**	0.001**	< 0.001***
Intermediate care unit [days]	0.0 (0.0–0.0)	5.0 (0.0–23.0)	0.0 (0.0–4.8)	0.0 (0.0–5.0)	0.041*	*0.245*	*0.095*
Intensive care unit [days]	0.0 (0.0–0.0)	1.0 (0.0–1.0)	0.0 (0.0–1.0)	0.0 (0.0–1.0)	0.033*	*0.059*	0.027*
Postoperative complications [Clavien-Dindo classification]							
Grade I	0 (0.0%)	0 (0.0%)	4 (28.6%)	4 (23.5%)	*1.000*	0.026*	*0.059*
Grade II	0 (0.0%)	3 (60.0%)	3 (21.4%)	6 (35.3%)	0.001**	*0.058*	0.016*
Grade III^4^	0 (0.0%)	0 (0.0%)	6 (42.9%)	6 (35.3%)	*1.000*	0.004**	0.016*
Grade IV^5^	0 (0.0%)	2 (40.0%)	1 (7.1%)	2 (11.8%)	0.010*	*0.129*	*0.059*
Grade V	0 (0.0%)	0 (0.0%)	0 (0.0%)	0 (0.0%)	*1.000*	*1.000*	*1.000*

Data are presented as the median and interquartile range (Q1–Q3) or as count and percentage. A *p*-value < 0.05 was considered statistically significant and are highlighted in boldface. Concerning symbolism and higher orders of significance: *p* < 0.05 *, *p* < 0.01 **, *p* < 0.001 ***. ASA status, American Society of Anesthesiologists physical status classification system; IPMN, intraductal papillary mucinous neoplasm; CRP, C-reactive protein. ^1^Two patients suffered from medical and surgical complications, therefore both have been allocated into the medical as well as the surgical complication group; ^2^total measured intraoperatively; ^3^postoperative observation period; ^4^includes grades IIIa and IIIb; ^5^ includes grades IVa and IVb

### Structure and longitudinal evolution of the gut microbiome

**Fig. 2:**
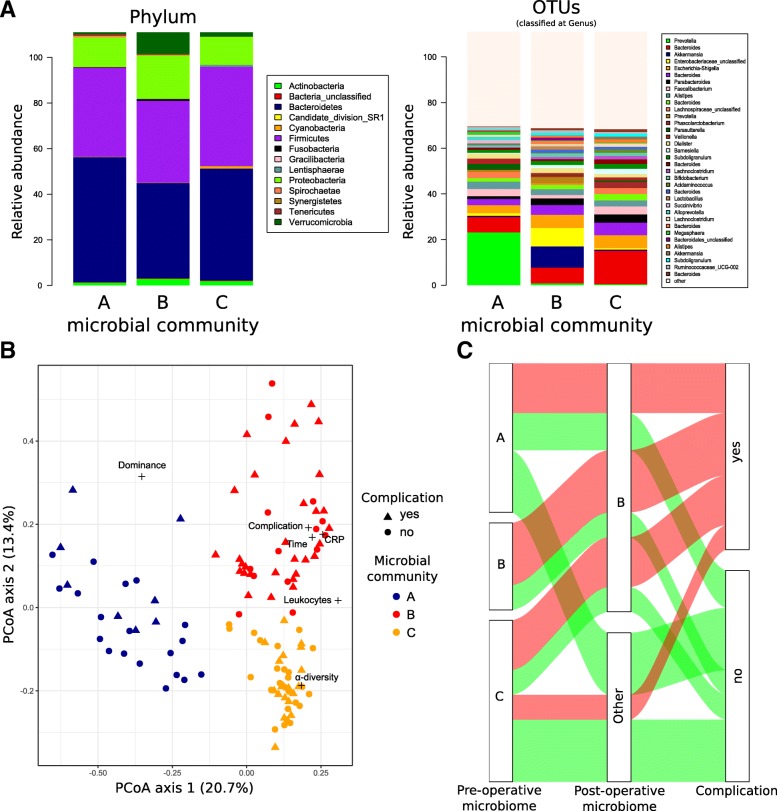
Microbial structure of the gut microbiome in the different microbial communities. **a** Microbial composition of the different microbial communities are represented at the phylum level on the left side and at the operational taxonomic unit (OTU) level on the right side. Only the 35 most abundant OTUs are represented. The remaining microbiota are included in the group “other”. **b** Microbial composition visualised by principal coordinate analysis (PCoA). Different colours correspond to the distinct microbial communities, where blue represents community A, red is community B and yellow is community C. Shapes correspond to the complication status, with circles representing no complication and triangles representing complications. Correlated clinical parameters and alpha-diversity indices are indicated by crosses. **c** Alluvial graph showing the evolution of the patients’ microbiome from the pre- to the post-operative period (group allocation based on colonisation with community B at least once during the distinct timespan) and association to the complication status

**Fig. 3:**
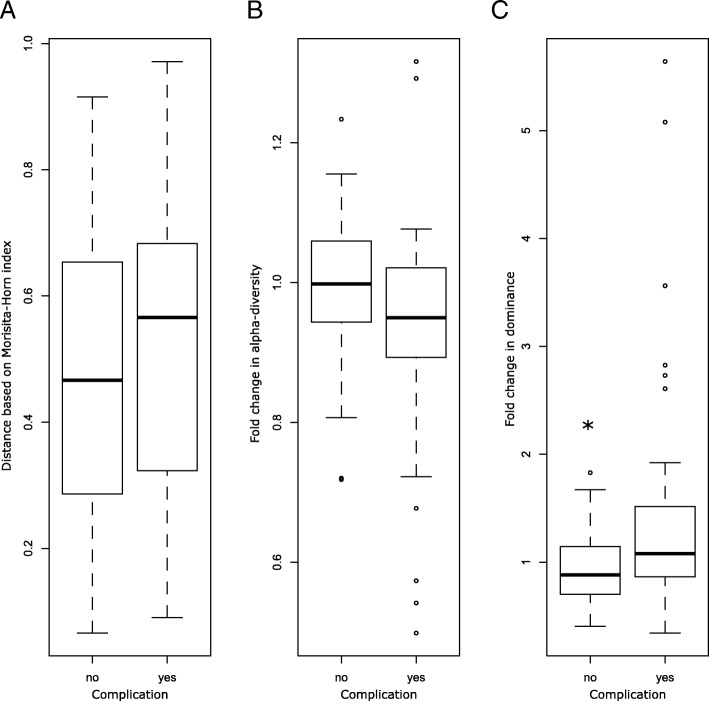
Longitudinal changes in the gut microbiome in regard to (**a**) distance, (**b**) alpha-diversity and (**c**) dominance between the pre- and post-operative microbiome in the non-complicated and in the complicated group

The gut microbiome in the studied cohort of patients was highly variable. The most abundant phyla in the dataset were *Bacteroidetes*, *Firmicutes* and *Proteobacteria*, and to a lesser extent, *Verrucomicrobia* and *Actinobacteria* **(**Fig. 2a). Based on the Morisita-Horn index, samples from the same patients were more closely related to each other than to samples from a different patient (Wilcoxon test, W = 184,400, *p* < 0.001***). We observed a significant correlation between clinical parameters and the PCoA axis, whereby the leucocyte count was positively correlated with axis 1, while the CRP level, the presence of a postoperative complication and the postoperative sample period were positively correlated with axes 1 and 2 (Fig. 2b). We observed that the majority of patients suffering from complications, showed microbial community B at least once during the postoperative period. As depicted in the alluvial graph, all patients presenting community B at the pre-operative time point continue to show a similar community during the postoperative period. Furthermore, the majority of patients with a complicated postoperative course harboured community B during the post-operation period (Fig. 2c). We did not observe any significant differences in species richness or alpha-diversity between samples from patients with or without postoperative complications. These results are consistent with those obtained using the PERMANOVA analysis indicating no significant change in the structure of the microbiome of samples from patients with or without postoperative complications. Nonetheless, the longitudinal evolution of the gut microbiome indicated a trend toward slight dysbiosis in the complicated cohort (Fig. 3).

### Distinct microbial communities of the gut microbiome

**Fig. 4:**
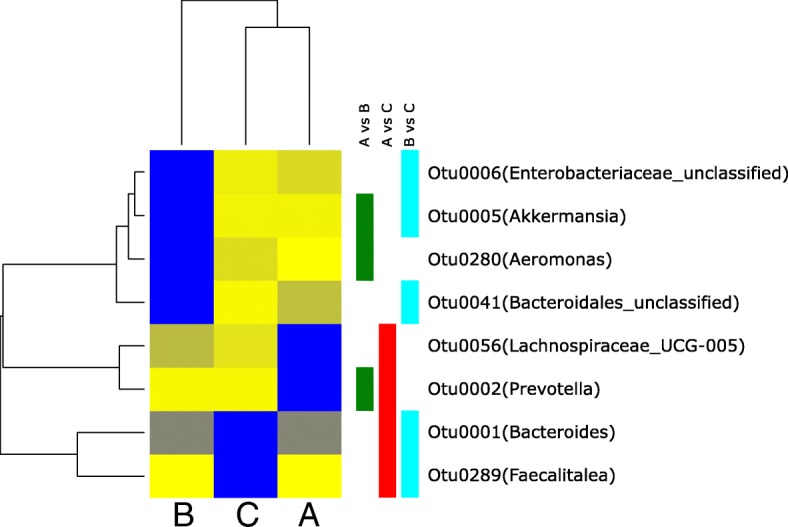
Differential abundance of each operational taxonomic unit (OTU) analysed by negative binomial distribution, represented in a heatmap for significant differential abundant OTUs. The mean abundance was normalised within each OTU to the maximal values. The colour code is from yellow (low abundance, 0) to blue (maximal abundance, 1). Green rectangles represent a significant differential abundance between microbial communities A and B, red squares represent a significant differential abundance between microbial communities A and C, and cyan squares represent a significant differential abundance between microbial communities B and C. The OTUs are named according to their genus classification

**Fig. 5:**
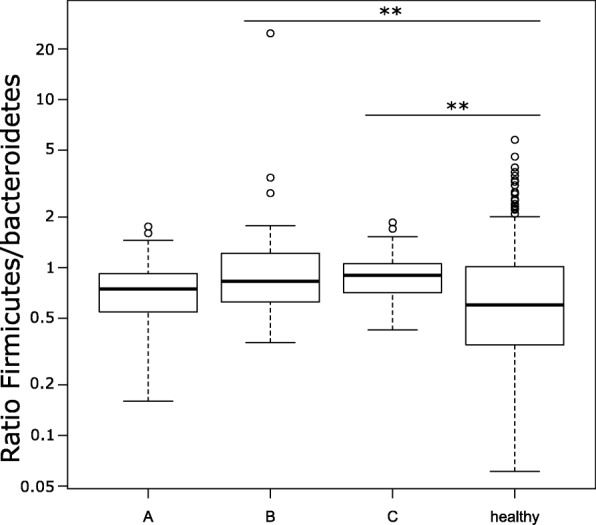
The *Firmicutes*/*Bacteroidetes* ratio in our cohort was compared to 235 healthy volunteers for whom V4 amplicon was available on the human microbiome project (HMP) database. The *Firmicutes*/*Bacteroidetes* ratio was calculated based on the read counts

Clustering analysis using the partitioning around medoid method indicated that based on the microbiome structure three microbial communities best described the cohort, namely community A, B and C **(**Fig. 2a**)**. Community B was composed of samples from the upper right side of the PCoA, whereas A and C clustered to the right and bottom left of the PCoA, respectively **(**Fig. 2b**)**. These communities did not show significant differences in regard to alpha-diversity and richness, but were hallmarked by a different microbial structure **(**Fig. 2a**)**. The only significant differences between community A and C were increased *Prevotella* and *Lachnospiraceae* in community A and increased *Bacteroides* and *Faecatitalea* in community C. Community B displayed an increase in *Akkermansia*, *Aeromonas*, *Enterobacteriaceae* and *Bacteroidales* and a decrease in *Lachnospiraceae*, *Prevotella, Faecatitalea* and *Bacteroides* **(**Fig. 4**)**. At the phylum level, only community B and C showed a moderate but significant (*p* < 0.01**) structural difference in the ratio of *Firmicutes/Bacteroidetes* (F/B ratio) compared to healthy volunteers **(**Fig. 5**)**.

### Microbial community B is associated with a higher complication rate

Patients with community B in the pre- and/or postoperative period showed no significant differences in their clinical baseline characteristics prior to surgery (primary disease, age, ASA status and cardiovascular risk factors) as well as perioperative factors (surgery time and blood loss) compared to those patients that showed another composition of microbial community. Partial pancreaticoduodenectomy was the only type of operation that was more frequent in patients with community B **(**Table 2**)**. Accordingly, samples belonging to community B were more often associated with bowel resection surgical procedures (B vs. A, odds ratio = 5.47, *p* < 0.01**; B vs. C, odds ratio = 2.96, *p* = 0.035*). Nevertheless, we did not observe any association between a specific type of operation and development of complications (odds ratio = 1.09, *p* = 1.000).

**Table 2:** Patients’ characteristics grouped by colonisation status (non-community B vs. community B)

	Non-community B	Community B	*p*-value non- community B. vs. community B
Number	13	19	
Age [years]	62.0 (51.0–68.0)	67.0 (58.0–75.0)	*0.150*
ASA status	2.0 (2.0–2.0)	3.0 (2.0–3.0)	*0.070*
Cardiovascular risk factors			
Coronary heart disease	1 (7.7%)	2 (10.5%)	*0.787*
Diabetes	3 (23.1%)	5 (26.3%)	*0.835*
Arterial hypertension	5 (38.5%)	8 (42.1%)	*0.837*
Chronic renal failure	0 (0.0%)	0 (0.0%)	*1.000*
Primary disease			
Malignant	6 (46.2%)	13 (68.4%)	*0.208*
IPMN	3 (23.1%)	5 (26.3%)	*0.835*
Chronic pancreatitis	4 (30.8%)	1 (5.2%)	*0.051*
Operation			
Partial pancreaticoduodenectomy	0 (0.0%)	7 (36.8%)	0.013*
Pylorus-preserving pancreas head resection	5 (38.0%)	6 (31.6%)	*0.687*
Total pancreatectomy	2 (15.4%)	1 (5.3%)	*0.335*
Distal pancreatectomy	3 (23.1%)	3 (15.8%)	*0.604*
Tumour enucleation	3 (23.1%)	2 (10.5%)	*0.337*
Surgery [min]	195.0 (180.0–285.0)	315.0 (210.0–345.0)	*0.273*
Blood loss^1^ [mL]	600.0 (200.0–1000.0)	700 (500–950)	*0.630*
Infusion^1^ [mL]	3000.0 (2500.0–4000.0)	3000.0 (2500.0–4000.0)	*0.684*
Transfusion^1^ [mL]	0.0 (0.0–0.0)	0.0 (0.0–0.0)	*1.000*
CRP^2^ [mg/L]	73.2 (36.1–105.1)	102.7 (51.6–155.0)	0.006**
Leucocytes^2^ [1/nL]	9.0 (7.8–9.9)	11.1 (8.4–13.8)	0.022*
Hospital stay			
In total [days]	11.0 (9.0–14.0)	15.0 (11.0–36.5)	0.030*
Intermediate care unit [days]	0.0 (0.0–0.0)	0.0 (0.0–3.0)	*0.439*
Intensive care unit [days]	0.0 (0.0–0.0)	0.0 (0.0–1.0)	0.032*
Postoperative complications [Clavien-Dindo classification]			
Total number	3 (23.1%)	14 (73.7%)	0.005**
Grade I	2 (15.4%)	2 (10.5%)	*0.683*
Grade II	1 (7.7%)	5 (26.3%)	*0.185*
Grade III^3^	0 (0.0%)	6 (31.6%)	0.044*
Grade IV^4^	0 (0.0%)	2 (10.5%)	*0.227*
Grade V	0 (0.0%)	0 (0.0%)	*1.000*
Outcome			
Survivor 90 days	13 (100.0%)	19 (100.0%)	*1.000*

Data are presented as the median and interquartile range (Q1–Q3) or as count and percentage. A *p*-value < 0.05 was considered statistically significant and are highlighted in boldface. Concerning symbolism and higher orders of significance: *p* < 0.05 *, *p* < 0.01 **, *p* < 0.001 ***. ASA status, American Society of Anesthesiologists physical status classification system; IPMN, intraductal papillary mucinous neoplasm; CRP, C-reactive protein. ^1^Two patients suffered from medical and surgical complications, therefore both have been allocated into the medical as well as the surgical complication group; ^2^total measured intraoperatively; ^3^postoperative observation period; ^4^includes grades IIIa and IIIb; ^5^includes grades IVaand IVb

**Fig. 6:**
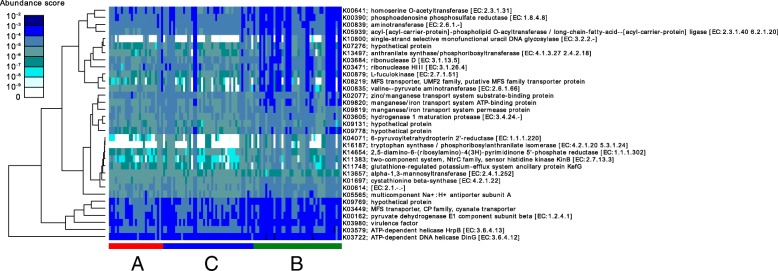
Heatmap showing the abundance score of the predicted function based on the taxonomical profile produced using Tax4Fun

Patients with community B showed significantly higher CRP levels and leucocyte counts in the postoperative period, as well as a significantly prolonged hospital stay and increased requirement for ICU treatment **(**Table 2**)**. Furthermore, samples from community B were more frequently associated with postoperative complications (B vs. A, odds ratio = 4.96, *p* < 0.01**; B vs. C, odds ratio = 2.89, *p* = 0.019*) and we observed a positive association between patients harbouring community B at least once during the study period and the development of a complication (odds ratio = 8.60, *p* < 0.01**; Fig. 2c). However, an occurrence of community B prior to surgery was not mandatory for the development of postoperative complications. Patients harbouring community B only during the postoperative period also showed increased complication rates as well as the same clinical characteristics as those patients presenting community B prior to surgery (e.g., age, ASA status, CRP levels, leucocyte count and hospital stay; Table 3). The predicted functions of the microbiota in community B were associated with significantly increased metabolism and transporter activity. We also observed an increase in a few functions involving the secretion system, the prokaryotic defence system and bacterial toxins **(**Fig. 6**)**.

**Table 3:** Patients’ characteristics grouped by colonisation status (non- community B vs. community B) and subdivided by the time-point of occurrence (pre- and/or postoperative period)

	Pre and post community B	Post community B	*p-value pre and post community B* vs. *post community B*
Number	7	12	
Age [years]	64.0 (58.5–67.0)	70.0 (58.5–75.0)	*0.445*
ASA status	2.0 (2.0–2.5)	3.0 (2.0–3.0)	*0.118*
Cardiovascular risk factors			
Coronary heart disease	0 (0.0%)	2 (16.7%)	*0.253*
Diabetes	2 (28.6%)	3 (25.0%)	*0.865*
Arterial hypertension	2 (28.6%)	6 (50.0%)	*0.361*
Chronic renal failure	0 (0.0%)	0 (0.0%)	*1.000*
Primary disease			
Malignant	4 (57.1%)	9 (75.0%)	*0.419*
IPMN	3 (42.9%)	2 (16.7%)	*0.211*
Chronic pancreatitis	0 (0.0%)	1 (8.3%)	*0.433*
Operation			
Partial pancreaticoduodenectomy	4 (57.1%)	3 (25.0%)	*0.161*
Pylorus-preserving pancreas head resection	1 (14.3%)	5 (41.7%)	*0.216*
Total pancreatectomy	1 (14.3%)	0 (0.0%)	*0.179*
Distal pancreatectomy	1 (14.3%)	2 (16.7%)	*0.891*
Tumour enucleation	0 (0.0%)	2 (16.7%)	*0.253*
Surgery [min]	330.0 (315.0–360.0)	240.0 (176.3–333.8)	*0.149*
Blood loss^1^ [mL]	750.0 (600.0–1300.0)	600.0 (500.0–800.0)	*0.251*
Infusion^1^ [mL]	3000.0 (2750.0–4250.0)	3000.0 (2375.0–3750.0)	*0.491*
Transfusion^1^ [mL]	0.0 (0.0–0.0)	0.0 (0.0–0.0)	*1.000*
CRP^2^ [mg/L]	147.5 (79.2–175.8)	86.7 (45.5–148.0)	*0.178*
Leucocytes^2^ [1/nL]	12.5 (8.6–14.0)	10.4 (8.0–12.9)	*0.370*
Hospital stay			
In total [days]	15.0 (10.5–32.0)	16.0 (11.8–33.8)	*0.641*
Intermediate care unit [days]	0.0 (0.0–5.5)	0.0 (0.0–0.3)	*0.539*
Intensive care unit [days]	1.0 (0.0–1.5)	0.0 (0.0–1.0)	*0.295*
Postoperative complications [Clavien-Dindo classification]			
Grade I	3 (42.9%)	2 (16.7%)	*0.211*
Grade II	1 (14.3%)	5 (41.7%)	*0.363*
Grade III^3^	0 (0.0%)	2 (16.7%)	*0.253*
Grade IV^4^	0 (0.0%)	0 (0.0%)	*1.000*
Grade V	1 (14.3%)	1 (8.3%)	*0.683*
Outcome			
Survivor 90 days	13 (100.0%)	19 (100.0%)	*1.000*

Data are presented as the median and interquartile range (Q1–Q3) or as count and percentage. A *p*-value < 0.05 was considered statistically significant and are highlighted in boldface. Concerning symbolism and higher orders of significance: *p* < 0.05 *, *p* < 0.01 **, *p* < 0.001 ***. ASA status, American Society of Anesthesiologists physical status classification system; IPMN, intraductal papillary mucinous neoplasm; CRP, C-reactive protein. ^1^total measured intraoperatively; ^2^postoperative observation period; ^3^includes grades IIIa and IIIb; ^4^includes grades IVa and IVb

### Primary disease

The postoperative histological results revealed in 19 patients a malignant pancreatic tumour, in 8 patients an intraductal papillary mucinous neoplasm (IPMN) and in 5 patients signs of a chronic pancreatitis. The incidence of postoperative complications did not differ significantly between the malignant and the IPMN group. In the chronic pancreatitis group no postoperative complications occurred **(**Table 1**)**. The primary disease seemed to have no influence on the pre- and postoperative microbial communities **(**Tables 2, 3**)**. Additional file 1: Table S1 gives also a more detailed overview about the several tumour stages and the occurrence of postoperative complications and the incidence of community B.

## Discussion

The composition of the gut microbiome, respectively the included members of the microbial community, play an important role by interacting with pathogens and preventing them from crossing the intestinal barrier [4–6]. Recent studies have established that the gut microbiome is dramatically altered in critically ill patients, in particular, decreased bacterial diversity during their stay in the ICU. This stress situation not only led to dysbiosis, but also the rampant growth of pathogens [10–16]. Therefore, the present study aimed to examine changes in the gut microbiome and the potential influence on the postoperative course in patients undergoing pancreatic surgery.

We were able to discriminate three separate microbial communities of the gut microbiome, each with a very different composition. All patients harboring community B already at the pre-operative period were also carrying this community during the post-operative period indicating a high stability and resistance of this microbiome type. Furthermore, community B was also associated with higher CRP levels, an increased leukocyte count, more frequent incidence of postoperative complications as well as prolonged hospitalisation. Interestingly, this was still the case when a patient was found to harbour community B only once during the observation period.

Based on the findings of previous studies with critically ill patients, we expected significant differences with regard to the alpha-diversity and richness in patients undergoing pancreatic surgery. Surprisingly, we did not observe this dramatic effect in our cohort of patients. Also, in the complicated group, gut microbiome patterns showed only slight changes toward dysbiosis. Both groups revealed a typical gut microbiome composition despite various external factors, such as systemic antibiotic use during surgery, indicating a stable and healthy gut microbiome [18]. However, there were large individual differences in the composition of the gut microbiome among patients at baseline (prior to surgery), as well as over the longitudinal course. Some patients showed large changes in the structure of their microbiome during the postoperative period, whereas others changed only marginally. This is due to the fact that patients previously bearing community B were not undergoing major changes in their microbiome structure while the two other communities were more prone to changes in their composition. Moreover, we were able to show that different samples from the same patient were more similar to each other than they were in comparison to samples from other patients. These results suggest that individual patients not only have a unique microbiome, but also react in a unique way to exposure to surgical stress and antibiotics. Therefore, we conclude that changes in richness and abundance, in addition to the overall microbiome structure, were factors correlated with a complicated clinical course.

Previous studies have suggested that the ratio of *Firmicutes/Bacteroidetes* (F/B-ratio) can be used as a surrogate marker for the outcome of critically ill ICU patients [10, 12]. The changes in F/B ratio in the current study were not as pronounced as those reported in previous studies of critically ill patients, which is probably due to our patient group being more homogeneous and having less severe diseases compared to those previously reported [10]. Therefore, its suitability to serve as a universal surrogate for patient outcome needs to be critically evaluated.

It has not yet been established why community B is associated with higher postoperative complication rates. Although alpha-diversity and richness were not significantly different among the distinct microbial communities, the specific composition at the OTU level was found to be different. The composition of community A was characterized by a high abundance of *Prevotella,* while community C showed an increase in *Bacteroides*. Based on the results of Arumugam et al., community A resembles the previous described enterotype 2 and community C resembles enterotype 1 [19]. Interestingly, the composition of community B differs from these previous reported results. The composition of community B was characterised, among others, by an increase in *Akkermansia* that belongs to the *Verrucomicrobia* phylum which degrade intestinal gut mucin as their sole carbon and nitrogen source [20]. The mucin layer of the intestinal gut has an important function as it acts as a physical barrier to protect epithelial cells from pathogen invasion [21, 22]. Animal experiments have shown a rise in mucin production, along with an increase in *Akkermansia* in the gut microbiome under pro-inflammatory conditions [23] and a protective effect on the epithelium [24, 25]. The increase in *Enterobacteriaceae,* which are usually located in the small intestine, remains unclear. However, this phenomenon has recently been described for patients undergoing Roux-en-Y gastric bypass operation due to acidity changes in the bowel milieu [5]. In contrast *Lachnospiraecae* were decreased, normally associated with resistance against colonisation by more pathogenic bacteria including *Clostridium difficile* [26], which might indicate an increased susceptibility of the intestinal flora.

Using Tax4Fun software to predict the potential functions of the gut microbiome in the different communities, significant increases in metabolism and transporter activity were observed which is consistent with the above-mentioned changes in community B. Moreover, an increase in functions involved with the secretion system, prokaryotic defence system and bacterial toxins was observed in community B. The results of this predictive analysis and the changes in the gut microbiome may indicate a shift toward a more competitive and virulent microbial community. However, it remains unclear whether the described changes represent a defence mechanism of the human immune system to prevent the invasion of microbes [27], or whether the changes themselves cause this problem. Furthermore, these functions are only prediction based on 16S profiles and should be validated by metagenomic or metatranscriptomic analyses to confirm the link between virulence factors and increased metabolism in community B. Nevertheless, our results reveal a clear connection between the respective community of the gut microbiome and the probability of developing a postoperative complication. Accordingly, implementation of a NGS-based approach for the detection of changes in the gut microbiome should be considered in routine diagnostics. In case of a detrimental microbial community, and assuming that changes in the gut microbiome are a cause rather than a consequence of postoperative complications, the avoidance of microbiome altering medications (i.e. antibiotics) and a combined individualized diet or in cases of non-response a stool transplantation might represent a causal therapeutic option. Where appropriate, this could be performed in the preoperative setting to modify the microbiome composition in order to lower the incidence of postoperative complications.

There are several limitations that need to be addressed in relation to the presented manuscript.

The previous described model of three several “enterotypes” by *Arumugam* et al. or the “microbial communities” presented in the recent paper, have to be considered with great caution. Because every patient will show a unique composition of its microbial structure within a community or “enterotype” and reacts in an individual way to external factors. This implies, that we may not recognize smaller but relevant changes in the individual microbial community or tend to overestimate them. The same holds true for the predicted functions, based on 16S profiles. 16S based microbiome studies are limited to describe changes in the abundance of the different taxa and can only estimate potential functions. Furthermore, no metabolomics or specific cellular functions were determined. The study was conducted in a single centre with a relatively small patient cohort, which only included patients undergoing pancreatic surgery. The correlation of the clinical complication rates and the changes in the gut microbiome might be influenced by heterogeneous types of complications, primary diseases, as well as different types of operations. Moreover, we are unable to make any conclusions regarding long-term patient survival, as the study was designed for a period of 90 days.

## Conclusion

In this study, we showed that differences in the gut microbiome are associated with the development of postoperative complications. Thus, sequencing of the gut microbiome or methods that take into account differences in the NGS-defined microbial communities might represent a useful diagnostic tool in future clinical practice. Whether the observed changes in the gut microbiome are a physiological defence mechanism or represent the cause remains unclear, and this needs to be evaluated in further investigations.

## Methods

### Study design

This prospective, observational, clinical study was approved by the Ethics Committee of the Medical Faculty of Heidelberg (trial code no. S-119/2015; German clinical trials register DRKS00008535). The study participants signed written informed consent. From October 2015 to August 2016, patients undergoing pancreatic surgery were screened for eligibility for study participation. Patients with an intake of antibiotics < 6 months prior to the operation, minors, pregnant women, allergy to antibiotics, and autoimmune or inflammatory intestinal diseases were excluded from the study. In total, 32 patients met the criteria and were enrolled in the study. A preoperative sample was collected to determine the baseline microbiome without surgical stress or antibiotic use, and at least two postoperative samples were taken to evaluate the longitudinal changes in the first 10 days after the operation. All stool samples were obtained by spontaneous defecation, without any manual stimulation. Whenever complications occurred that led to prolonged hospitalisation, further samples were taken up to 30 days after the operation, to assess changes in the gut microbiome associated with the complicated clinical course. All patients were re-evaluated for survival 90 days after the operation. Each patient received a standardised single shot of 1 g sulbactam and 2 g ampicillin 30 min before the surgical procedure. Postoperatively, the participating patients were classified into two groups consisting of those who suffered postoperative complications and those who had a non-complicated clinical course. The complication group was further subdivided into patients who suffered from surgical (e.g., bleeding, anastomotic insufficiency or pancreatic fistula) or medical (e.g., pneumonia or lung artery embolism) complications. Postoperative complications were classified according to the Clavien-Dindo classification [28, 29].

### Collection and storage of stool samples

In total, 116 stool samples from 32 patients were collected and snap frozen in liquid nitrogen. Afterwards the samples were stored at − 80 °C until further processing. Deoxyribonucleic acid (DNA) extraction was performed from 200-mg stool samples using the PSP® Spin Stool DNA Kit (Stratec, Birkenfeld, Germany) according to the manufacturer’s protocol (number 3). This ensured isolation of total DNA from all bacteria including those that are difficult to lyse. Negative controls involved performing the extraction without any clinical sample.

### Microbiome analysis

Polymerase chain reaction (PCR) was performed on total DNA using universal bacterial primers flanking the V4 region (515F and 806R) [30], including negative controls (from sterile water to evaluate contamination from the PCR reagent and the negative control processed during extraction to control carry-over from the reagent in the extraction kit) to exclude contamination [31]. Sequencing adapters were ligated to the PCR products and paired-end sequenced using an Illumina Miseq system (250 cycles). The raw sequences obtained from sequencing were checked to remove low-quality sequences and chimera. To normalise the sampling effort, sequences were subsampled to the same number of reads per sample. Operational taxonomic units (OTU) were defined using a threshold of 3% divergence, and representative sequences were classified at the taxonomic level by comparison with the SILVA database (version 128) using Mothur software [32]. More than 9 million good-quality non-chimeric reads were obtained, which were subsampled to 1110 reads per sample (Good’s coverage of 97.3%, range 94.6–99.6%). Sequencing of a mock community of known species allowed us to calculate the overall error rate of the PCR and sequencing methodologies. The error rate was 1.89 × 10^− 5^ errors per base. Sequences data are deposited in figshare (https://figshare.com/s/8420f9d19f0bbfa2c3f3).

### Statistical analysis

Descriptive indices for alpha-diversity (non-parametric Shannon index), richness (Chao1 richness estimate), dominance (relative abundance of the most abundant OTU) and evenness (Shannon index-based measure of evenness) were calculated from the OTU table. Variation in alpha-diversity was tested by a pairwise Wilcoxon sum rank test. Beta-diversity variation was evaluated via principal coordinate analysis (PCoA) and PERMANOVA based on Morisita-Horn similarity index [33]. Based on the PCoA distribution, cluster analysis was performed to define the microbial communities using the ‘partitioning around medoids’ algorithm [34]. We used Tax4Fun software to predict the potential function present in each sample based on the taxonomic composition [35]. We used the package DAtest (Russel et al. 2018) to compare different differential abundance methods and used the more accurate method (based on AUC, FDR and FPR) to compare our groups. The best method was an analysis with a model based on a negative-binomial distribution (DESeq2) to detect differences in the abundance of OTUs between the groups [36]. All statistical analyses were performed with MOTHUR 1.37.4 and R 3.3.0 software. A *p*-value of < 0.05 was considered statistically significant.

The clinical data were entered into an electronic database (Excel 2011; Microsoft Corp., Redmond, WA, USA) and analysed using SPSS software (version 24.0; SPSS Inc., Chicago, IL, USA). Categorical data were summarised using absolute and relative frequencies. Quantitative data were summarised using the median and quartiles. The Kolmogorov-Smirnov test was applied to check for normal distribution. In the case of non-normally distributed data, non-parametric methods were used for evaluation (chi-square test for categorical data, Mann-Whitney U test for continuous data). Correlation analyses were performed by calculating the Spearman rank correlation coefficient (Spearman’s rho/ρ). A *p*-value of < 0.05 was considered statistically significant. Concerning symbols used to represent higher orders of significance, *p* < 0.05 was indicated by *, *p* < 0.01 by ** and *p* < 0.001 by ***.

## Additional file

Additional file 1: **Table S1.** Patients with a malignant pancreatic tumour (*n* = 19), grouped by the Union for International Cancer Control (UICC)-Stage (Stage: I-IV) and subdivided by colonisation status (community B) or postoperative complications. (DOCX 54 kb)
