# Supplementary material for: Gut microbiome patterns correlate with higher postoperative complication rates after pancreatic surgery
Source: BMC Microbiol. 2019 Feb 18;19:42. doi: 10.1186/s12866-019-1399-5 (PMC6379976; doi:10.1186/s12866-019-1399-5)
Supplement: Supplementary file 1 — Table S1. Patients with a malignant pancreatic tumour (n = 19), grouped by the Union for International Cancer Control (UICC)-Stage (Stage: I-IV) and subdivided by colonisation status (community B) or postoperative complications. (DOCX 54 kb) [file 12866_2019_1399_MOESM1_ESM.docx]

**Additional file 1: Table S1.**

| **UICC - Stage** | **Patients**  **(n=19)** | **community B**  **(n=9)** | ***p-value***  ***UICC - Stage vs. community B*** |
| --- | --- | --- | --- |
| **Stage 0** | 3 | 1 (11.1%) | *0.948* |
| **Stage I** | 2 | 2 (22.2%) | *0.274* |
| **Stage II** | 10 | 6 (66.7%) | *0.537* |
| **Stage III** | 1 | 0 (0.0%) | *0.162* |
| **Stage IV** | 3 | 0 (0.0%) | *0.162* |
| Data are presented as count and percentage. A p-value <0.05 was considered statistically significant. Concerning symbolism and higher orders of significance: p < 0.05 *, p < 0.01 **, p < 0.001 ***. UICC-Stage, Union for International Cancer Control (UICC)-Stage | | | |

| **UICC - Stage** | **Patients**  **(n=19)** | **complicated**  **(n=10)** | ***p-value***  ***UICC - Stage vs. complicated*** |
| --- | --- | --- | --- |
| **Stage 0** | 3 | 1 (10.0%) | *0.531* |
| **Stage I** | 2 | 1 (10.0%) | *1.000* |
| **Stage II** | 10 | 5 (50.0%) | *0.835* |
| **Stage III** | 1 | 0 (0.0%) | *0.305* |
| **Stage IV** | 3 | 3 (30.0%) | *0.060* |
| Data are presented as count and percentage. A p-value <0.05 was considered statistically significant. Concerning symbolism and higher orders of significance: p < 0.05 *, p < 0.01 **, p < 0.001 ***. UICC-Stage, Union for International Cancer Control (UICC)-Stage | | | |
